# The Spatio-Temporal Distribution Patterns of Biting Midges of the Genus *Culicoides* in Salta Province, Argentina

**DOI:** 10.1673/031.012.14501

**Published:** 2012-12-11

**Authors:** Cecilia A. Veggiani Aybar, María J. Dantur Juri, Mirta Santana, Mercedes S. Lizarralde de Grosso, Gustavo R. Spinelli

**Affiliations:** ^1^ Instituto Superior de Entomología “Dr. Abraham Willink”, Facultad de Ciencias Naturales e lnstituto Miguel Lillo, Universidad Nacional de Tucumán, Miguel Lillo 205, (4000) Tucumán, Argentina; ^2^ IAMRA, Universidad Nacional de Chilecito, 9 de Julio N° 22, (F5360CKB) Chilecito, La Rioja, Argentina; ^3^ Cátedra de Bioestadística, Facultad de Medicina, Universidad Nacional de Tucumán, Lamadrid 875, (4000) Tucumán, Argentina; ^4^ División Entomología, Museo de La Plata, Paseo del Bosque s/n, (1900) La Plata, Buenos Aires, Argentina

**Keywords:** abundance, Ceratopogonidae, climatic variables, Diptera

## Abstract

The goal of this survey was to analyze the spatio-temporal distribution patterns of *Culicoides* Latreille species (Diptera: Ceratopogonidae) and their relationship with environmental variables in Salta, northwestern Argentina. *Culicoides* were collected monthly from January 2003 through December 2005. The influence of the climatic variables on population abundance was analyzed with a multilevel Poisson regression. A total of 918 specimens belonging to five species were collected. The most abundant species was *Culicoides paraensis* Goeldi (65.5%), followed by *Culicoides lahillei* Iches (14.6%) and *Culicoides debilipalpis* Lutz (7.6%). The highest seasonal abundance for *C. paraensis*, *C. debilipalpis* and *C. lahillei* occurred during the spring and summer. A Poisson regression analysis showed that the mean maximum and minimum temperature and the mean maximum and minimum humidity were the variables with the greatest influence on the population abundance of *Culicoides* species.

## Introduction

The genus *Culicoides* Latreille (Diptera: Ceratopogonidae) is known to include vectors of different pathogens of humans and animals throughout the world (Borkent and Spinelli 2007). More than 1,200 species worldwide are included in this genus, of which 266 species have been reported for the Neotropical region (Borkent and Spinelli 2007). Fifty-one *Culicoides* species have been reported for Argentina, of which 21 were reported in the northeast (Spinelli et al. 2005), such as *Culicoides insignis* Lutz, *Culicoides venezuelensis* Ortiz and Mirsa, *Culicoides leopoldoi* Ortiz, *Culicoides limai* Barretto, *Culicoides flinti* Wirth, *Culicoides debilipalpis* Lutz, *Culicoides paraensis* Goeldi, and *Culicoides guttatus* Coquillett. In the northwest of the country, six species have been reported: *Culicoides crescentis* Wirth and Blanton, *C. debilipalpis*, *Culicoides lahillei* Iches, *C. insignis*, *C. paraensis*, and *C. venezuelensis* (Veggiani Aybaret al. 2010).

The health importance of certain *Culicoides* species is the result not only of the discomfort caused by the insects’ bites but also of their role as transmitters of disease. These species transmit filariasis, Oropouche virus, bluetongue virus, and African horse sickness virus (Mellor et al. 2000; Ronderos et al. 2003). In addition, the bites can cause immediate or delayed reactions that range from allergic dermatitis, papules, and pustules as a result of overinfection caused by scratching, to more severe reactions such as eczema, desquamation, and scars with alterations in skin pigmentation (Sherlock and Guitton 1965; Kettle 1995).

In northwestern Argentina, research has only been focused on the transmission of filariasis in humans. This disease is caused by the nematode *Mansonella ozzardi* (Manson) (Biglieri and Aráoz 1915; Mühlens et al. 1925; Romaña and Wygodzinsky 1950; Remondegui et al. 1988; Taranto and Castelli 1988; Shelley and Coscaron 2001). Its vectors include certain *Culicoides* species, i.e., *C. paraensis* and *C. debilipalpis* in Tucumán province (Romaña and Wigodzinsky 1950) and *C. lahillei* and *C. paraensis* in Jujuy province (Shelley and Coscaron 2001).

Despite its importance as a vector of *M. ozzardi*, little is known about the distribution and abundance of *Culicoides* in northwestern Argentina, as demonstrated by the few published works on the subject. Thus, the main objective of this work was to identify the *Culicoides* species present in the subtropical mountainous rainforest of Salta and to determine not only the spatial and temporal distribution patterns of *Culicoides* species, but also the influence of climatic variables on the population abundance of these species.

## Materials and Methods

### Study area

**Figure 1.**
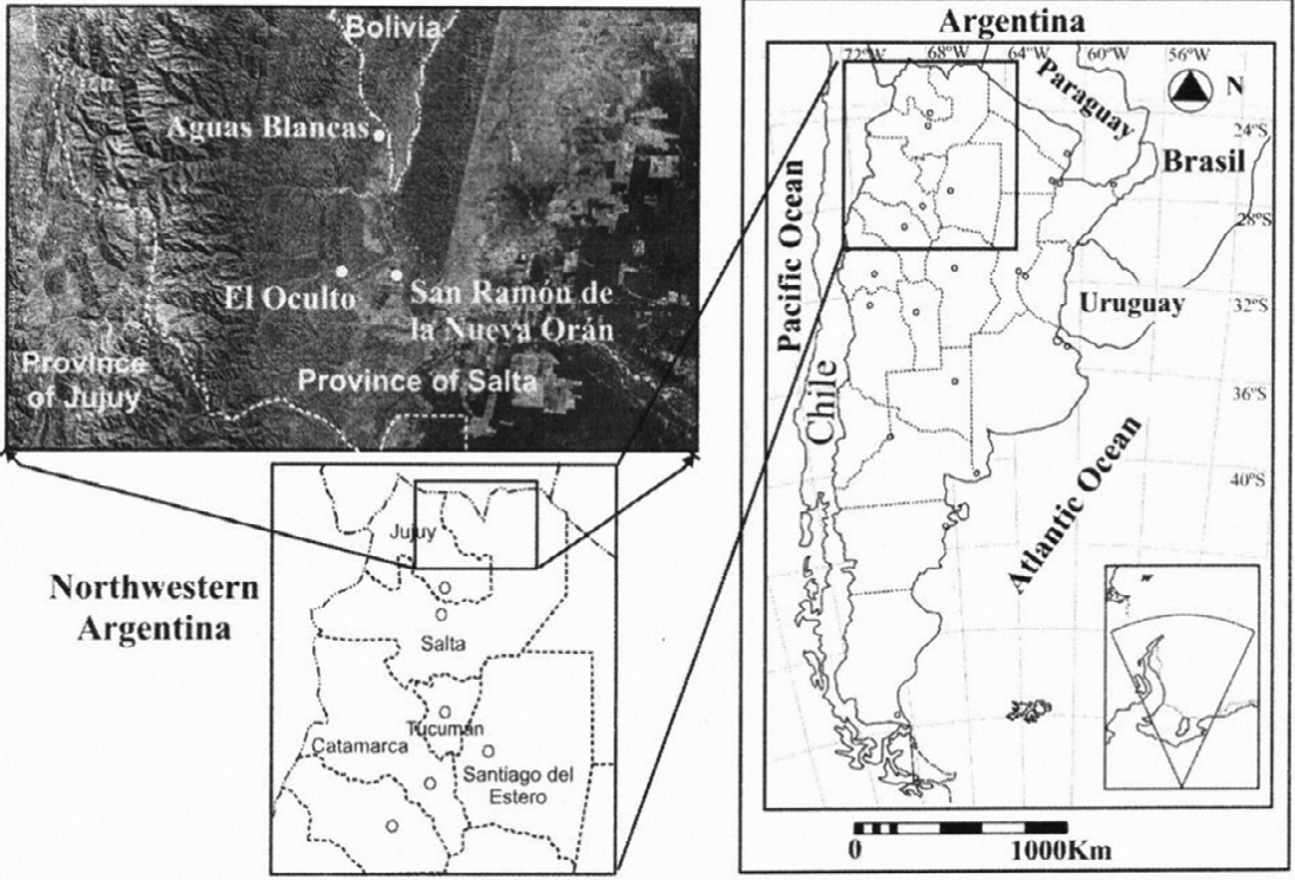
Geographical locations of Aguas Blancas, El Oculto, and San Ramón de la Nueva Orán, Salta province, northwestern Argentina. High quality figures are available online.

The study was conducted in three localities of the Orán department in northern Salta province, located between 22° 33′ to 24° 17′ S and 63° 24′ to 65° 04′ W. The localities selected were Aguas Blancas (22° 43′ S, 64° 22′ W), El Oculto (23° 06′ S, 64° 30′ W), and San Ramón de la Nueva Orán (23° 08′ S, 64° 20′ W). These sites are located in the forest of “palo blanco and palo amarillo”within the subtropical mountainous rainforest (Cabrera 1976) (Figure 1).

The climate of the area is subtropical with a dry season. The monthly mean temperatures range between 13° C and 30° C, and the annual mean temperature is 21.4° C. The summers are hot (21° C to 35° C) and wet (78% relative humidity), with extreme temperatures of up to 45° C. The winters are mild (8.3° C to 24° C). The average annual rainfall is 734 mm, with a maximum in January (157 mm) and a minimum between June and August (4 mm). The area has a monsoon rainfall regime, and the maximum rainfall coincides with the maximum temperatures (Arroyo 2004).

### Collection of specimens

During the development of a research project based on the bioecological and parasitological aspects of *Anopheles* mosquitoes found in northwestern Argentina, specimens of *Culicoides* were collected monthly using CDC light traps baited with carbon dioxide. The traps were active for two consecutive days from 17:00 to 23:00 (during the sunset and early evening hours). A total of four traps were used at each site. The traps were placed on the branches of trees at a height of 1.20 m above the ground at a distance of 100 m from one another.

The climatic variables considered for the study were the mean maximum and minimum temperature, mean temperature, mean maximum and minimum humidity, mean humidity, accumulated rainfall, and wind velocity. All these variables were recorded by the meteorological stations of San Ramón de la Nueva Orán (23° 07′ 60″ S, 64° 19′ 60″ W) and Aguas Blancas (22° 43′ 60″ S, 64° 22′ 00″ W).

### Processing and identification of *Culicoides* specimens

The specimens collected were preserved in 70% ethanol and subsequently identified with the taxonomic keys of Spinelli et al. (2005) and Borkent and Spinelli (2007). Some specimens were processed using microscopic techniques for the mounting of specimens according to the phenol-balsam method of Wirth and Marston (1968). The use of these mounted specimens facilitated the correct identification and/or verification of the species. Voucher specimens were deposited in the collection of the Miguel Lillo Foundation Institute (Instituto-Fundación Miguel Lillo-IMLA).

### Data analysis

The influence of the climatic variables on the population abundance of the different species of *Culicoides* was analyzed with a multilevel Poisson regression (Statacorp 2005). The analysis was performed with ULM6 software using penalized quasi-likelihood. Generally, this method is used for longitudinal studies in which the observations are not mutually independent because the response variable is measured several times for the same individual (Twisk 2004). In the present study, the method was used to model the effect of the climatic variables on the abundance of *Culicoides* for a three-year sampling period.

The analysis considers two levels: level 1, whose units were months, and level 2, whose units were years. In level 1, the climatic variables were expressed as values centered on the annual mean (*X _ijk_*).

The level 1 linear structural model was:
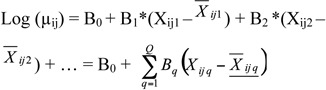

where µ_ij_ is the event rate per period of time, X_ijq_ is a level 1 predictor, B_0_ is the intercept, B_q_ represents the level 1 slope for the independent predictor variable X_ijq_, and Q is the level 1 number of predictor variables.

The model for level 2 was:
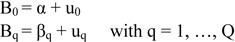

where α and β_q_ are the fixed effects and u0 and Uq the random effects. The random effects indicate the variability of the level 2 coefficient B_q_.

In this case, the model best suited for reliable estimation was obtained with a random intercept. Thus, the level 2 model was:
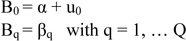

The first level 2 equation indicates that the incidence rate changes according to the year independently of the values of the climatic variables, whereas the second level 2 equation indicates that the relationship between the incidence rate and the climatic variables does not change according to the year.

One of the results obtained with this analysis, the incidence rate ratio (IRR), is extremely important because it shows the degree of influence of each variable on the number of specimens collected. Additional statistical parameters were obtained, including the standard error, the value of *p* (*p* < 0.001) and the confidence interval (95%).

## Results

### Abundance and seasonal variation of *Culicoides*

A total of 918 *Culicoides* specimens were collected in the subtropical mountainous rainforest of Salta province. Of these specimens, 65.5% belonged to *C. paraensis*, followed by *C. lahillei* (14.6%), *C. debilipalpis* (7.6%), *C. insignis* (2.4%), and *C. venezuelensis* (0.7%) (Table 1).

**Table 1.**
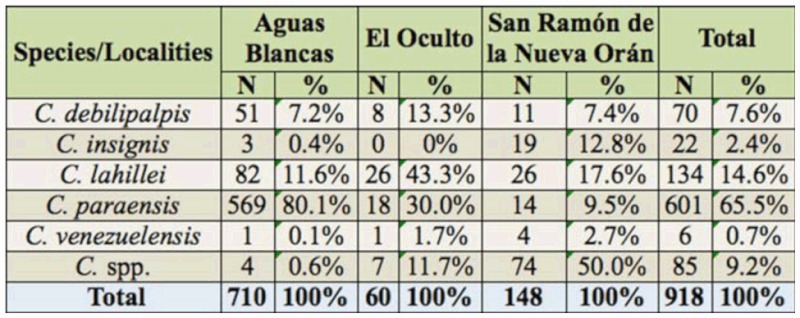
Absolute number (N) and percentage (%) of *Culicoides* species collected from January 2003 through December 2005 in Aguas Blancas, El Oculto and San Ramón de la Nueva Orán, Salta province, Argentina.

The abundance of *Culicoides* per sample site showed that Aguas Blancas was the locality with the greatest number of specimens (77.4%), followed by San Ramón de la Nueva Orán (16.1%) and El Oculto (6.5%).

**Figure 2.**
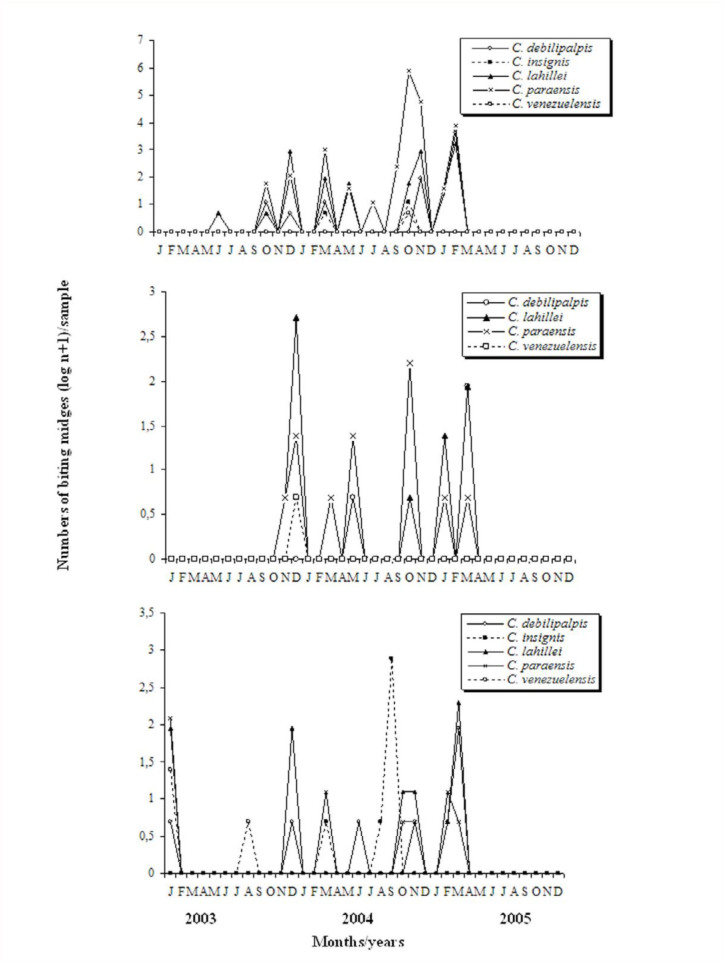
Seasonal fluctuations of *Culicoides debilipalpis*, *Culicoides insignis*, *Culicoides lahillei*, *Culicoides paraensis*, and *Culicoides venezuelensis* from January 2003 through December 2005 in Aguas Blancas, El Oculto, and San Ramón de la Nueva Orán, Salta province, Argentina. High quality figures are available online.

The analysis of the seasonal distribution of the specimens showed that a different pattern was found for each of the *Culicoides* species in each of the localities sampled. At Aguas Blancas, *C. paraensis* was abundant during the spring (October to November) and summer (December), whereas both *C. debilipalpis* and *C. lahillei* were abundant during the summer (December to February). At El Oculto, *C. lahillei* showed peaks of abundance during the summer (December) and fall (March), whereas *C. paraensis* showed peaks during the spring (October) and *C. debilipalpis* during the fall (March). At San Ramón de la Nueva Orán, *C. lahillei* was abundant during the summer (December to January) and fall (March), *C. paraensis* during the summer (January), and *C. insignis* during the spring (September) (Figure 2).

### Effects of the climatic variables

The effects of the climatic variables on the population abundance of *Culicoides* showed a characteristic pattern for each of the species collected. In general, the mean maximum and minimum temperature and mean maximum and minimum humidity were the variables that exerted the greatest influence on the abundances of the species. For *C. debilipalpis*, both the mean maximum temperature and mean maximum humidity were significant (*p* < 0.0001 in each case). According to the IRR, the abundance of this species increased 12% for each 1° C increase in the mean maximum temperature, whereas the abundance increased 19% for each 1% increase in the mean maximum humidity (Table 2). For *C. paraensis*, the mean maximum temperature and mean maximum humidity were the significant variables (*p* < 0.0001 in each case). The IRR showed that the abundance of this species increased 16% if the mean maximum temperature increased 1° C, whereas it decreased 0.8% if the mean maximum humidity increased 1% (Table 2). *C. insignis* was influenced only by the mean minimum humidity (*p* < 0.0001). According to the IRR, the abundance of this species decreases 0.9% if the mean minimum humidity increases 1% (Table 2). For *C. lahillei*, the mean minimum temperature was the only significant variable (*p* < 0.011). For each 1° C of increase in the mean minimum temperature, the abundance of this species increased 24% (Table 2). The low number of specimens of *C. venezuelensis* collected made statistical analysis impossible.

**Table 2.**
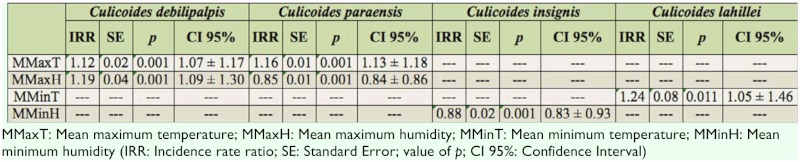
Multilevel Poisson regression results for *Culicoides debilipalpis*, *Culicoides paraensis*, *Culicoides insignis*, and *Culicoides lahillei* species collected from January 2003 through December 2005 in Salta province, Argentina.

## Discussion

A relatively detailed understanding of the behavior of *Culicoides* species in nature is necessary for the design and implementation of vector control strategies. This study presents the results of a preliminary survey of the spatio-temporal distribution patterns of several species of the genus *Culicoides* in Salta province, northwestern Argentina. Five species of *Culicoides* were identified. Three of these species, *C. debilipalpis*, *C. lahillei*, and *C. paraensis*, were previously reported from the area (Romaña and Wigodzinsky 1950; Shelley and Coscaron 2001), and two, *C. insignis* and *C. venezuelensis*, were recently reported from Salta province (Veggiani Aybar et al. 2012). Note that the latter two species were recently reported for Tucumán, in northwestern Argentina (Veggiani Aybar et al. 2010).

Although relatively few specimens were collected during this study, the data suggest that the most abundant species were *C. paraensis*, *C. lahillei*, and *C. debilipalpis*. Similar results were reported from Tucumán province by Veggiani Aybar et al. (2010), who found that *C. paraensis* was the most abundant species. Ronderos et al. (2003), in a study conducted in the area of the Yacyretá Dam between Argentina and Paraguay, reported *C. paraensis* as a very common and widely distributed species and *C. lahillei* as a species that was collected occasionally.

Mercer et al. (2003) also reported that *C. paraensis* was the most abundant species, both in peri-urban and urban areas in Iquitos, Perú. The presence and abundance of *C. paraensis* in the study area could be consistent with the suggestion of De Barros et al. (2007) that because this species is anthropophilic, it would show a substantial capacity to survive and reproduce in human environments. This characteristic of the species would explain its broad geographical range, which extends from the United States to Argentina. The relatively high abundance of *C. paraensis* found in the present study is of interest because this species is a known secondary vector of filariasis and was found naturally infected with *M. ozzardi* in the subtropical mountainous rainforest of northwestern Argentina, where this disease is endemic (Shelley and Coscaron 2001).

Two species whose abundance was less than that of *C. paraensis* are of particular interest. *C. lahillei* is considered to be of great importance because it is the principal vector involved in the transmission of *M. ozzardi* (Shelley and Coscaron 2001). *C. debilipalpis* has been identified as a secondary vector of this nematode in northwestern Argentina (Romaña and Wigodzinsky 1950).

During the present study, specimens of *Culicoides* were collected in larger or smaller numbers each month. They were found during all four seasons in Aguas Blancas and San Ramón de la Nueva Orán, but not in El Oculto. These results agree with the findings of Sabio (2005) and De Barros et al. (2007), who found specimens in the USA and Brazil during the entire year.

The greatest population peaks for *C. paraensis*, *C. debilipalpis*, and *C. lahillei* occurred during the spring and summer. The abundance of these species then gradually decreased, and these species almost disappeared during the winter. Our results are consistent with the findings of Sabio (2005), who found that the greatest abundance of *C. paraensis* and *C. debilipalpis* in Louisiana (USA) occurred during the spring. Both species were also abundant during the fall, and the former species was also abundant during the winter. Our results are also consistent with the findings of Veggiani Aybar et al. (2010) that in Tucumán province, *C. paraensis*, *C. lahillei*, and *C. insignis* were abundant in the summer, whereas *C. debilipalpis* was abundant during the fall and (to a lesser extent) during the spring.

The mean maximum and minimum temperature and the mean maximum and minimum humidity were the significant climatic variables in this study. An increase in the mean maximum humidity was associated with an increase in the abundance of *C. debilipalpis*, whereas an increase in the mean maximum temperature was associated with an increase in the abundance of *C. debilipalpis* and *C. paraensis*. An increase in the mean minimum temperature was associated with an increase in the abundance of *C. lahilleii* whereas decreases in the abundance of *C. paraensis* and *C. insignis* were associated with increases in the mean maximum humidity and mean minimum humidity, respectively. Consequently, it could be inferred that the influence of these variables may have created an environment favorable for the species to fluctuate in different ways and to be abundant at different times of the year. This pattern of influence was cited by Kay and Lennon (1982), who found that temperature and humidity were the most important climatic variables in the determination of the seasonality patterns of *Culicoides* species. In addition, several authors have reported the direct relationship between temperature, rainfall, and humidity on the population abundance of *Culicoides* in Brazil, due to the influence of these climatic variables on the life cycles of the species or on alterations in their breeding places (Sherlock and Guitton 1964; Santos da Silva et al. 2001; De Barros et al. 2007). In Argentina, Veggiani Aybar et al. (2010) cited accumulated rainfall as the climatic variable most strongly related to the abundance of *Culicoides* species. They also cited relative humidity, mean temperature, and wind speed as significant factors.

Despite the substantial progress achieved by the present research, much remains to be learned about the biology and behavior of *Culicoides* in the subtropical mountainous rainforest of northwestern Argentina. Future research will focus on specific collection methods and methodologies (traps baited with UV light and trapping hours from dusk to dawn) that would contribute to a better understanding of the population dynamics of *Culicoides* in northwestern Argentina.

## Abbreviations

IRR,: incidence rate ration
